# Food Stuck in the Throat in a Newly Diagnosed Diabetes Mellitus Patient: An Atypical Presentation of Wallenberg’s Syndrome

**DOI:** 10.7759/cureus.38076

**Published:** 2023-04-24

**Authors:** Pooja Roy, Okeoghene Akpoigbe, Abiodun M Akanmode, Comfort Anim-Koranteng, Rahman Olusoji

**Affiliations:** 1 Internal Medicine, Harlem Hospital Center, New York, USA

**Keywords:** wallenberg’s syndrome, diabetes mellitus, dysphagia, lateral medullary syndrome, posterior inferior cerebellar syndrome

## Abstract

Wallenberg’s syndrome, also known as posterior inferior cerebellar artery syndrome (lateral medullary syndrome), is known to be a common cause of posterior ischemic stroke syndromes in men in their 60s and may present with varied symptoms devoid of focal neurological signs making it easily missed as a differential of posterior ischemic strokes. It involves a stroke in the vertebral or posterior inferior cerebellar artery of the brainstem. In this case report, we critically examine the case of a 66-year-old man with newly diagnosed diabetes whose main presentation was dysphagia and unsteady gait. There was no motor or sensory examination finding in our patient, and the initial computed tomography of the brain was negative for any intracranial pathology leading to very low suspicion of stroke. However, given a high index of suspicion and a thorough oropharyngeal examination ruling out structural abnormality, magnetic resonance imaging of the brain revealed features suggestive of Wallenberg’s syndrome. This case emphasizes careful consideration of posterior stroke syndrome when evaluating patients presenting with dysphagia without typical motor/sensory symptoms of cerebrovascular accident and the requirement of further imaging to support the diagnosis.

## Introduction

Wallenberg’s syndrome is also referred to as the posterior inferior cerebellar artery syndrome or the lateral medullary syndrome. The earliest description of the features of this syndrome was in 1810. Vasper Viesseux at the Medical and Surgical Society of London described the features as “vertigo, unilateral facial numbness, loss of pain and temperature appreciation in the opposite limbs, dysphasia and hoarseness, minor tongue involvement, hiccups (cured by taking up the habit of a morning cigarette) and a drooped eyelid” [1].

However, in a case report by Wallenberg in 1895, he expanded on the clinical manifestations and supported this by the precise localization of the lesion, as evidenced by his findings from autopsy specimens. He published four case reports carefully detailing and highlighting the features of what we now refer to as Wallenberg’s syndrome [1].

Anatomically, the vertebral arteries originate from the subclavian artery and the posterior inferior cerebellar artery (PICA) which provides arterial supply to the medulla and the suboccipital aspect of the cerebellum. Hence, the neurologic manifestation of Wallenberg’s syndrome is secondary to damage in the lateral segment of the medulla posterior to the inferior olivary nucleus, with atherosclerotic occlusion of the vertebral artery accounting for the most common cause, followed by the occlusion of the PICA, and the least common is the vertebral arteries [2,3]. Posterior ischemic strokes account for 20% of all ischemic strokes. Hypertension, diabetes, and smoking are high-risk factors associated with the development of Wallenberg’s syndrome due to their role in atherosclerosis [2-4]. Wallenberg’s syndrome accounts for the bulk of posterior ischemic stroke syndromes, with men typically in their 60s mostly affected [2,3].

The clinical manifestations of Wallenberg’s syndrome tend to vary remarkably [4-7]. Typically, patients present with vertigo with nystagmus (typically central and beating in the direction of the gaze), truncal ataxia, Horner syndrome (ptosis, anhidrosis, and miosis), ipsilateral incoordination of extremities, impaired superficial sensation to the ipsilateral side of the face and contralateral limb and trunk, and ipsilateral palsy of the soft palate, larynx and pharynx (giving rise to dysarthria, dysphonia, dysphagia and the associated loss of gag reflex). In atypical patients, isolated lateropulsion or vestibular symptoms have been noted [3-5]. In this case report, we highlight the case of a 66-year-old male with features suggestive of Wallenberg’s syndrome with an atypical presentation.

## Case presentation

A 66-year-old male from the Dominican Republic with no known medical history presented to the emergency department because of generalized weakness and difficulty swallowing for four days. He first noticed his symptoms after he felt food stuck in his throat, describing dysphagia as both solid and liquid with difficulty swallowing the saliva that started all of a sudden with a choking sensation. He noticed difficulty in deglutition causing increased spitting and provoking cough when attempting to swallow. He denied nausea, vomiting, odynophagia, chest pain, previous history of similar symptoms, fever, night sweats, or any vertigo.

He also reported progressive generalized weakness for four months before presentation, which was associated with polyuria, polyphagia, polydipsia, nocturia, and undocumented weight loss. He was not a known diabetic and denied any family history of diabetes. He reported being unsteady on his feet after the onset of dysphagia and reported a funny feeling and had been cautious to prevent falls. He denied dysarthria, facial weakness, visual changes, or loss of sensation in any part of the body. He was a non-smoker with no history of alcohol or illicit drug use.

On arrival at the emergency department, he was afebrile with a temperature of 98.2°C, respiratory rate of 17 breaths per minute, blood pressure of 143/84 mmHg, and oxygen saturation of 95% on room air. On physical examination, his mucous membrane was dry; head, ears, eyes, nose, and throat examination showed an absent gag reflex; and dystaxia with a tendency more to the left side of the body. Moreover, he was found to have unprovoked hiccups on the first day of his admission. A complete neurological examination was done. He was alert and oriented to time, place, and person and fluent in language with good comprehension. On cranial nerve examination, only loss of gag reflex was appreciated. The motor examination revealed normal muscle bulk and tone. Strength was 5/5 in all four extremities both proximally and distally. Intact fine motor movements bilaterally. There was no pronator drift. The sensation was intact to light touch, pinprick, vibration, and proprioception throughout, and the Romberg sign was negative with normal reflex in the upper and lower extremity. Cerebellar signs were preserved. However, the gait was wide-based ataxic. The rest of the examination, including cardiovascular, respiratory, and abdominal, showed no abnormalities.

**Figure 1:**
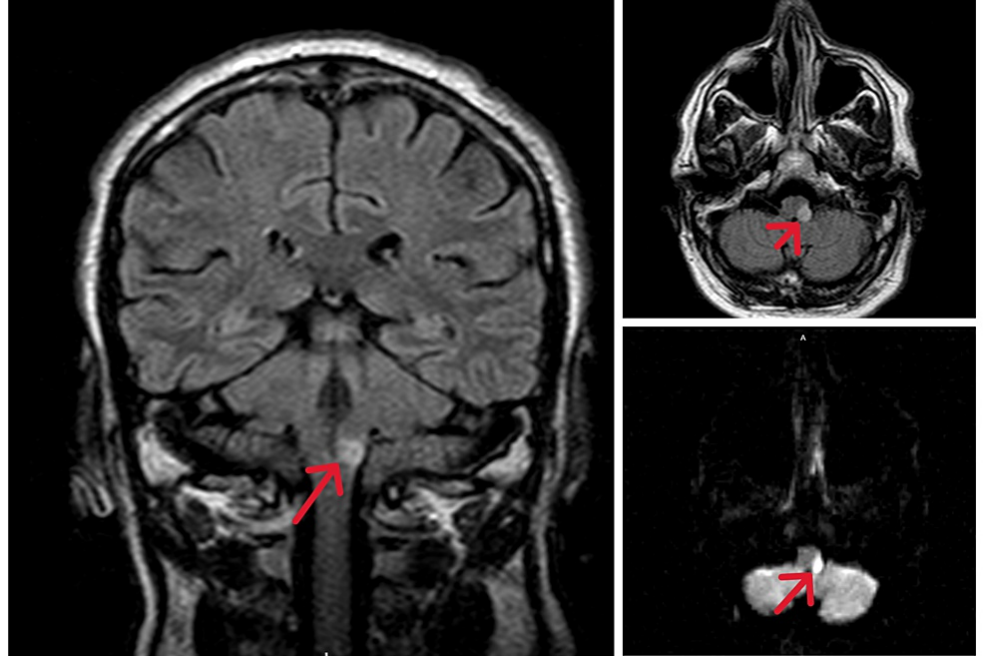
MRI of the brain FLAIR imaging (first picture: coronal and left upper axial) showing restricted diffusion in the left superior posterolateral medulla (red arrow) consistent with ischemia associated with DWI (lower left) and high signal in this region with mass effect, suggestive of posterior inferior cerebellar artery infarct. MRI: magnetic resonance imaging; FLAIR: fluid-attenuated inversion recovery; DWI: diffusion-weighted imaging

The computed tomography (CT) of the head initially showed no acute intracranial abnormality. For the evaluation of dysphagia after consulting with otolaryngology, a CT scan of the soft tissue of the neck without contrast did not identify any foreign body or any external compression. CT angiography of the head and neck showed no evidence of hemodynamically significant stenosis, dissection, occlusion, or aneurysm. Therefore, magnetic resonance imaging (MRI) of the brain without contrast was done, which revealed an area of restricted diffusion in the left superior posterolateral medulla consistent with ischemia associated with T2/fluid-attenuated inversion recovery (FLAIR) high signal in the same region, suggestive of acute-to-subacute infarct (Figure 1). He was diagnosed with lateral medullary syndrome and was started on aspirin 81 mg daily, clopidogrel 75 mg daily, and Lipitor 80 mg nightly. For the newly diagnosed diabetes, he was started on metformin 500 mg once daily. He was further evaluated for his neurologic dysphagia by flexible laryngoscopy which revealed a normal nasal cavity, nasopharynx, oropharynx, supraglottis, and glottis; however, left vocal cord paralysis was noted and was considered a part of the neurological deficit. He was unable to swallow both solid and liquid or thick food. Due to a high risk of aspiration, he was advised to undergo a temporary percutaneous endoscopic gastrostomy, but he denied the procedure despite multiple counseling and was discharged with a nasogastric tube instead with the physical medicine and rehabilitation and neurology outpatient follow-up.

On the repeat follow-up in the outpatient neurology clinic, repeat speech-language pathology evaluation, and physical therapy were continued and he was able to pass the swallow test with a low risk of aspiration. He continued to follow up as an outpatient with gradual improvement of his swallowing ability with increased oral intake.

## Discussion

Posterior circulation stroke (PCS) has varied symptoms and requires a high index of suspicion for diagnosis. Our patient, a 66-year-old male, presented with a sensation of food stuck in the throat associated with dysphagia and hoarseness for three days. Interestingly, the neurological examination was normal except for the absent gag reflex and ataxic gait. The usual symptoms of patients with PCS are dizziness, vertigo, gait instability, imbalance, and dysphagia [7-9]. Our patient had gait instability and dysphagia, suggesting the consideration of PCS as a differential.

Patients presenting with dysphagia should undergo a complete neurological assessment, including cranial and cerebellar testing, and any possibility of mass, infection, lymphadenopathy, or food bolus should be ruled out [10]. Neurological examinations such as bedside swallow tests, any contralateral deviation of the uvula or tongue, and loss of gag reflex aid in the diagnosis [8-10]. Romberg test and HINTS (head impulse, nystagmus, test of skew) can further narrow down the differentials [7,10]. However, lack of examination finding on the initial emergency visit may lead to keeping stroke in the least considered differentials. Furthermore, CT scans are inferior in finding the posterior strokes though they are the initial imaging of choice. Our patient started to develop hiccups on his first day of admission, which is reported to be caused by lesions affecting the dorsal nucleus of the vagus and nucleus of the solitary tract (pure sensory nuclei clusters forming gray matter nested in the medulla oblongata) and an imbalance between inspiration and expiration [5].

CT is known to have low specificity in identifying early infarcts in addition to low sensitivity in documenting posterior fossa infarcts, whereas MRI is effective in recognizing early infarcts with T2-weighted images as it is more sensitive than FLAIR [5-7]. Diffusion-weighted MRI is a widely used modality in identifying PCS [5,6]. Our patient had a negative initial CT scan which led us to proceed with MRI.

Patients with lateral medullary syndrome present with different symptoms due to the variance of damage to the nucleus. Infarct in the vestibular nucleus and vestibulo-cerebellar connection can present with vertigo with nystagmus and gastrointestinal symptoms, whereas defects in the nucleus ambiguus, glossopharyngeal, and vagus nerve may present with dysphonia, dysarthria, dysphasia found in 51-94% of cases along with loss of gag reflex and hiccups [5,6,10]. Involvement of cerebellar peduncles, spinocerebellar fibers, and inferior cerebellar hemisphere may present with ipsilateral ataxia in addition to ipsilateral Horner syndrome when sympathetic fibers are involved [6]. Loss of pain and thermal sensation on the ipsilateral face and contralateral trunk and limbs represents the impairment of the nucleus spinalis, trigeminal nerve, or spinothalamic tracts. Interestingly, more than 90% of patients will have some sensory impairment; however, our patient did not have sensory impairment on presentation, making it obscure to keep in the differentials [6].

## Conclusions

Typical cases of Wallenberg’s syndrome present with loss of pain and thermal sensation in association with motor symptoms such as unilateral paralysis or vertigo. This case exemplifies an atypical case of Wallenberg’s syndrome presenting with dysphagia which had been progressive for a few months without the usual sensory symptoms associated with Wallenberg’s syndrome. CT imaging studies were negative; however, on further evaluation with MRI due to his unsteady gait, he was found to have Wallenberg’s syndrome. The absence of any specific motor or sensory finding in a patient presenting with dysphagia might deviate the physician from considering Wallenberg’s syndrome.
